# Stroke Strikes in Silence: Bilateral Medial Medullary Infarction in a Patient With Complete Hearing Loss

**DOI:** 10.7759/cureus.52965

**Published:** 2024-01-25

**Authors:** Francisco J Gallegos Koyner, Fernando Amador, Nehemias A Guevara, Nelson Barrera, Salomon Chamay, David Chong

**Affiliations:** 1 Internal Medicine, St. Barnabas Hospital Health System, New York, USA; 2 Pulmonary and Critical Care Medicine, St. Barnabas Hospital Health System, New York, USA

**Keywords:** hearing impairment, neurology and critical care, drug-resistant hypertension, stroke, bilateral medial medullary infarction

## Abstract

Bilateral medial medullary infarction (BMMI) is a rare stroke subtype that accounts for less than 1% of acute strokes. Common manifestations of this stroke include quadriparesis, bilateral hypoglossal palsy, bilateral sensory loss, and respiratory failure. We present the case of a 39-year-old male with deafness and mutism who was brought to the emergency department due to acute onset of altered mental status and generalized weakness, further decompensated, and was lately diagnosed with bilateral medial medullary infarction. This case hopes to illustrate a differential diagnosis to be considered and promptly managed when a patient presents with altered mental status and quadriparesis, especially in the acute setting where tissue plasminogen activator (tPA) can still be given.

## Introduction

Medial medullary infarction (MMI) is a rare stroke subtype, and a bilateral presentation of this type of stroke is even rarer (<1% of all strokes) [1,2]. Suspecting this kind of stroke strongly relies on the neurologic exam, however, when a patient presents with altered mental status in the context of a coexisting hearing impairment, obtaining an adequate history and neurologic exam is difficult. In this instance, physicians may be less inclined to consider stroke as the likely cause for the presentation and take other differentials as their main, which might delay diagnosis. Our case highlights how this delay in diagnosis can reflect in poorer outcomes. 

This article was previously presented as a meeting abstract at the 2023 CHEST Annual Meeting on October 9, 2023. 

## Case presentation

We present the case of a 39-year-old male patient with a past medical history significant for resistant hypertension (R-HTN), deafness and mutism, heart failure with preserved ejection fraction, chronic kidney disease and multiple previous strokes who was brought to the emergency department (ED) due to acute onset of altered mental status (AMS) and quadriparesis. As per roommate, the patient rolled from the couch, had a fall and hit his head 2 days prior to presentation, and since then was acting strangely. 

**Figure 1:**
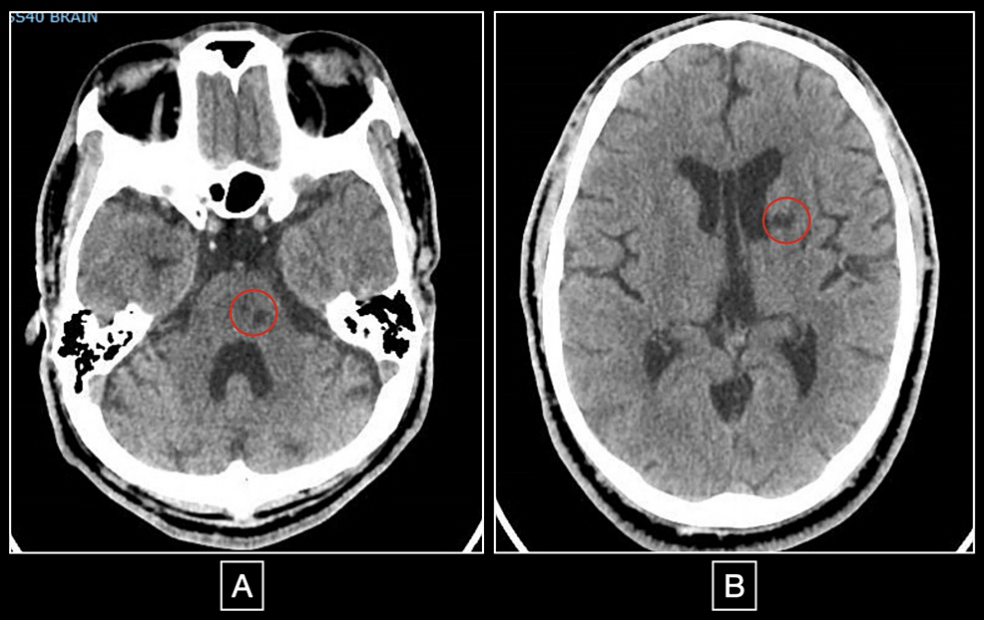
Non-contrast CT brain showing old infarcts These are 2 cuts of the non-contrast CT brain which was done in the emergency department. Figure A demonstrates a hypodensity in the left pons (red circle), which was later confirmed by MRI to be an old pontine stroke, and figure B shows a hypodensity in the left caudate nucleus (red circle) which is also consistent with an old stroke. This CT was negative for any acute pathology.

Initial vital signs were blood pressure of 204/129 mmHg, heart rate of 130, temperature of 100.2ºF, and respiratory rate of 22. The patient was interviewed with the aid of a sign-language translator, who stated that the patient appeared confused, but was able to follow commands. On physical examination, the patient was noted to be in distress, have generalized weakness (⅗ in bilateral upper extremities and ⅖ in bilateral lower extremities), and poor coordination. Deep tendon reflexes were normal. A 3/6 holosystolic murmur was heard in the aortic area, and bilateral crackles were heard on pulmonary auscultation. Initial CT of the brain showed old pontine and basal ganglia infarcts and was otherwise negative for an acute pathology (Figure 1). A lumbar puncture was done and was normal. Initial testing was significant for hypokalemia. Bilateral adrenal hyperplasia was noted on CT Abdomen/Pelvis, which was otherwise negative. A chest X-ray showed bilateral infiltrates.

At this moment, the decision was made to admit the patient, and the impression was of metabolic encephalopathy, hypertensive emergency, heart failure exacerbation and to rule out pneumonia. Generalized weakness was initially not interpreted as quadriparesis. During the first hours after admission, his weakness worsened, his AMS progressed (he was now noted to be oriented only in person), and he developed signs of bulbar dysfunction, such as an inability to manage secretions, aspiration of secretions, and dysphagia. Subsequently, the patient developed acute hypoxemic respiratory failure, initially requiring a non-rebreather mask, but later due to persistent tachypnea and inspiratory stridor, requiring intubation. 

**Figure 2:**
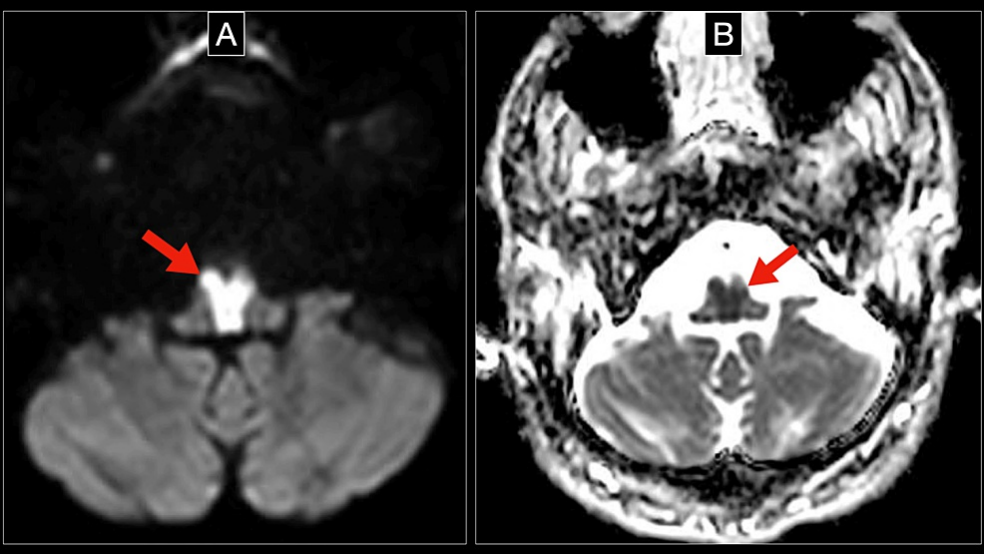
Brain MRI sequences showing an acute bilateral medial medullary infarction Both images are taken from the brain MRI done on the patient a few days after presentation. Figure A (image on the left) shows the DWI sequence at the level of the medulla, and figure B (image on the right) shows the ADC sequence at the same level, both showing the classic "heart sign" (red arrows), characteristic of a bilateral medial medullary infarction. ADC: apparent diffusion coefficient; DWI: diffuse weighted image

**Figure 3:**
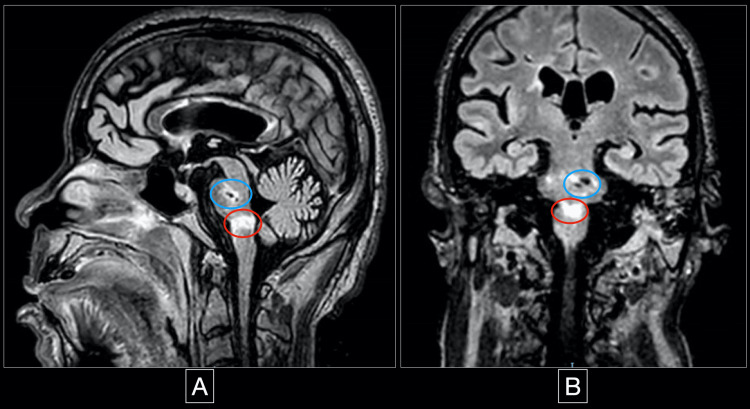
Sagittal and coronal cuts of the same brain MRI showing the acute stroke and encephalomalacia of the left pons Figure A is the sagittal view and figure B is the coronal view of the same brain MRI, this time on fluid-attenuated inversion recovery (FLAIR) sequence, and both of them show the acute medullary stroke in the red circle and a left pontine encephalomalacia consistent with an old stroke in the blue circle.

**Figure 4:**
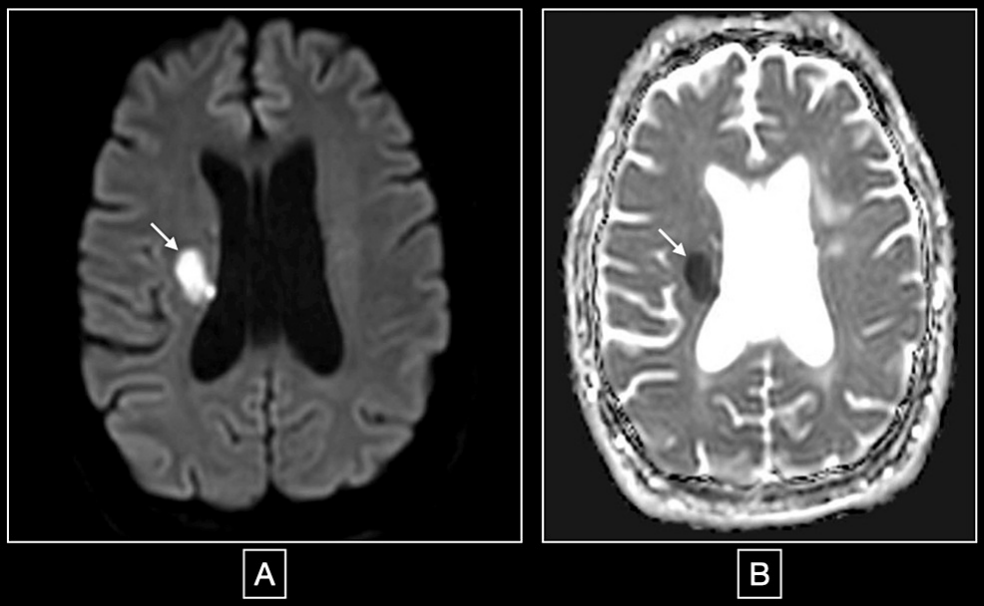
DWI and ADC sequences showing an acute insular stroke Figure A (image on the left) is the DWI sequence, and figure B (image on the right) is the ADC sequence of the MRI brain, and both show an acute right stroke of the subcortical insular lobe extending into the corona radiata (signaled by a white arrow). ADC: apparent diffusion coefficient; DWI: diffuse weighted image

Neurologic examination without sedation was remarkable for no response to pain, pupillary escape, nystagmic jerks, and normal direct reflex but no consensual reflex. Due to the constellation of findings, a brainstem lesion was suspected, and an MRI of the brain was performed. The MRI showed an acute bilateral medial medullary stroke with the classic “heart” sign, seen on diffuse weighted image (DWI) and apparent diffusion coefficient (ADC) (Figure 2, 3), in addition to a subcortical insular lobe stroke (Figure 4).

**Figure 5:**
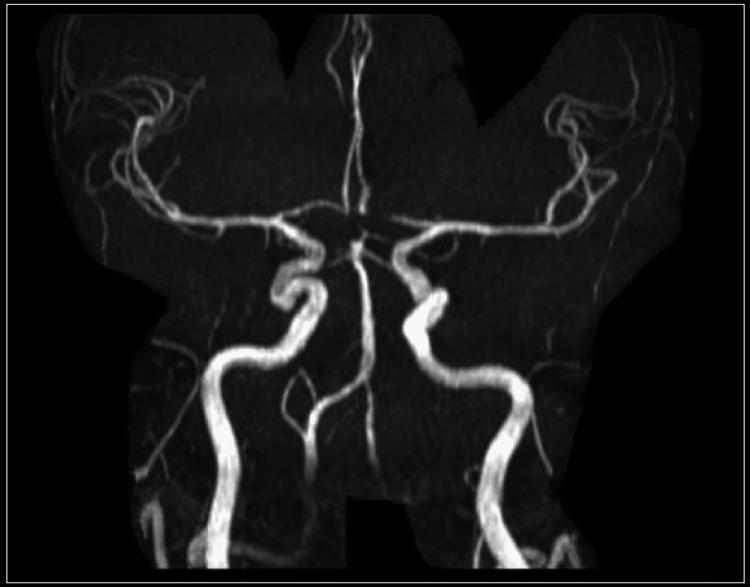
Magnetic resonance angiography (MRA) of the brain The MRA of the brain was reported as unremarkable.

Magnetic resonance angiography (MRA) of the brain did not show any significant pathology (Figure 5). The echocardiogram showed severe left ventricular hypertrophy, ejection fraction of 50%, mild calcification of the aortic valve, and grade II left ventricular diastolic dysfunction. Atrial fibrillation was not noted in telemetry. With this information and a history of multiple previous lacunar strokes, Neurology determined that the strokes were due to small-vessel disease. Cerebral autosomal dominant arteriopathy with subcortical infarcts and leukoencephalopathy (CADASIL) and cerebral autosomal-recessive arteriopathy with subcortical infarcts and leukoencephalopathy (CARASIL) were suspected as genetic etiologies for his multiple lacunar infarcts, but confirmatory genetic testing came back negative. 

The patient was worked up for causes of secondary hypertension due to adrenal hyperplasia noted on previous imaging. Laboratories were significant for high renin levels (24) and borderline high aldosterone levels (19); renal ultrasound showed elevated resistive indices of the bilateral renal arteries; however, his MRA of the abdomen showed normal caliber renal arteries. Further workup was not pursued due to poor prognosis. The patient did not recover from respiratory failure or quadriparesis, nor did his mental status improve; therefore, a tracheostomy and percutaneous gastrostomy were performed. After 5 months of initial presentation, the patient died. 

## Discussion

Bilateral medial medullary infarction (BMMI) is rare and it accounts for less than 1% of acute strokes. Unilateral MMI is the most common presentation and is comprised of the Dejarine triad of contralateral hemiparesis sparing the face, contralateral hemisensory loss, and ipsilateral hypoglossal involvement [1,3]. A bilateral presentation of MMI is less common and it may account for 20% of medial medullary infarctions. Comparably, BMMI may present clinically with quadriparesis, bilateral hypoglossal palsy, bilateral sensory loss, and respiratory failure [1,2].

Initial diagnosis is challenging owing to its heterogeneous presentation. The advent of brain MRIs has facilitated its diagnosis by demonstrating a “heart sign” in DWI at the rostral level of the medial medulla, figure 2A. This pattern has been described in several case reports and it has been considered pathognomonic for BMMI [4,5].

Guillain-Barre syndrome (GBS) and brainstem encephalitis may mimic BMMI, but their presentation is usually subacute; CSF analysis may provide some information to aid in the diagnosis [6], specifically for acute presentations of BMMI in which radiologists and clinicians may miss developing infarcts on brain MRIs [7].

Speculations of its origin may suggest a unilateral occlusion of the distal vertebral artery with an underlying anatomical variant of the anterior spinal artery (ASA) or the paramedian anterior spinal branches originating from a single vertebral artery. Others suggest an unpaired ASA as an anatomical variation. A bilateral thromboembolic occlusion of the branches of the anterior spinal artery has also been demonstrated. A normal MRA of the brain suggests small-vessel disease and cardioembolic origin should always be considered [5,8-10]. In our patient with a normal MRA of the brain, no evidence of atrial fibrillation or thrombus in the echocardiogram, multiple previous lacunar strokes, and the presentation of uncontrolled hypertension, small-vessel disease was concluded to be the cause of the infarction. 

The prognosis is generally worse than unilateral MMI. In a systematic review, most BMMI survivors remained dependent and one-fifth of patients died [2]. Our patient unfortunately didn’t recover and died 5 months after the initial presentation.

In our patient, quadriparesis, nystagmus, and respiratory failure correlated with DWI. Other neurological signs may have been blurred by underlying strokes, baseline mutism, and deafness. 

## Conclusions

Our case accentuates the importance of early clinical suspicion and diagnosis in medullary strokes, and how they may be masked due to certain comorbidities. The case serves as a sobering reminder of the need for accurate assessment in cases of acute neurological emergencies in an attempt to optimize patient outcomes, although most cases in the literature have had poor prognoses regardless. 
